# Twenty-four Hour Ambulatory Blood Pressure Monitoring in Open Angle Glaucoma Suspects: A cross-sectional descriptive study

**DOI:** 10.22336/rjo.2024.71

**Published:** 2024

**Authors:** Mahesh Bharathi, Subashini Kaliaperumal, Sandhiya Selvarajan, Renuka Srinivasan, Mary Stephen

**Affiliations:** 1 Glaucoma Consultant, Ahalia Eye Hospital, Palakkad, India; 2 Department of Ophthalmology, Jawaharlal Institute of Postgraduate Medical Education and Research (JIPMER), Puducherry, India; 3 Dept of Clinical Pharmacology, Jawaharlal Institute of Postgraduate Medical Education and Research (JIPMER), Puducherry, India; 4 Pondicherry Institute of Medical Sciences, Puducherry, India

**Keywords:** ambulatory blood pressure, glaucoma suspect, Retinal Nerve Fibre Layer, dippers, vascular, BP = Blood Pressure, SBP = Systolic Blood Pressure, DBP = Diastolic Blood Pressure, DPP = Diastolic Perfusion Pressure, ABP = Ambulatory Blood Pressure, MAP = Mean Arterial Pressure, OCT = Optical Coherence Tomography, RNFL = Retinal Nerve Fiber Layer, IOP = Intraocular pressure, POAG = Primary Open Angle Glaucoma

## Abstract

**Purpose:**

To compare glaucoma suspects’ 24-hour blood pressure pattern with healthy subjects and the Retinal Nerve Fibre Layer (RNFL) thickness among dippers and non-dippers.

**Materials and methods:**

We included 100 patients diagnosed as glaucoma suspects in the study group and 100 age and gender-matched controls. Twenty-four-hour ambulatory blood pressure (BP) was measured using an automated BP monitoring device for mean systolic BP (SBP), mean diastolic BP (DBP), and mean arterial pressure (MAP). We classified patients into non-dippers, dippers, and overt dippers based on reduction in nocturnal MAP. Structural damage to the optic nerve head was studied by measuring the superior, inferior, and average RNFL thickness on Optical Coherence Tomography (OCT).

**Results:**

Glaucoma suspects showed lower values of day, night, and mean SBP values but higher values of day, night, and mean DBP values when compared with controls, and these were statistically significant. ANOVA and Post Hoc test (Bonferroni) analysis among glaucoma suspects showed that overt dippers had statistically significant superior, inferior, and average RNFL thinning (average 86.20 ± 12.200 µm) as compared to non-dippers and dippers (average 105 ± 11.183 and 102.19 ± 9.582 µm respectively). Pearson’s correlation, used to assess the relationship between the nocturnal dip in BP and average RNFL thickness, showed a negative correlation (r = -0.396, p < 0.001).

**Discussion:**

Our study found a statistically significant decrease in systolic blood pressure day and night and an increase in diastolic blood pressure day and night in glaucoma suspects compared to normal. Mean arterial pressure did not show any significant difference, and the data obtained is comparable with previous studies. The corresponding retinal nerve fiber layer changes noted in dippers and non-dippers were also similar to those in the existing literature. This study, however, has a shortcoming of not including intraocular pressure-related nerve head changes.

**Conclusion:**

Nocturnal BP reduction was associated with structural damage to the optic nerve head in glaucoma suspects, suggesting systemic vascular etiology in the damage progression. ABP monitoring can help detect those glaucoma suspects who are mainly likely to progress so that they can be on close follow-up.

## Introduction

Globally, glaucoma continues to be the second leading cause of irreversible blindness, affecting around 3% of the population above the age of 40 years [1]. Among the various spectrum of glaucoma patients ranging from primary open-angle glaucoma (POAG) to angle closure glaucoma to various other secondary and complicated glaucomas, a subset of the population presents as the “glaucoma suspect”. Glaucoma suspect is a subgroup of the population with features suspicious of glaucomatous optic nerve damage and visual field damage but is not diagnostic of glaucoma. This group of people, if diagnosed earlier, can be followed up regularly as they are at risk of developing open-angle glaucoma. Their early diagnosis will also aid in deciding upon the plan of management and the need for prophylactic treatment.

Previous studies have shown a positive correlation between intraocular pressure (IOP) and systemic BP among open-angle glaucoma patients of various races [2-6]. This association is found to be more with diastolic blood pressure (DBP) than systolic blood pressure (SBP), and a higher frequency of glaucoma is noted in patients with low diastolic perfusion pressure (DPP) [3,4,7].

Studies on 24-hour ambulatory blood pressure (ABP) monitoring are few in glaucoma, with no conclusive evidence on the role of blood pressure. While few studies in normal tension glaucoma showed a more nocturnal dip in BP, others did not find any difference between patients with glaucoma and the general population [8,9]. However, some studies suggested that systemic hypotension, especially nocturnal hypotension, is a risk factor for glaucomatous optic nerve damage [10-12]. There are hardly any studies where ABP monitoring for 24-hour blood pressure patterns has been carried out in glaucoma suspects.

Retinal nerve fiber layer (RNFL) damage assessment is critical in assessing the structural damage to the optic nerve head as it manifests earlier than the functional damage, which appears in visual field defects. Hence, RNFL damage can help detect glaucoma and can be an indicator of early disease. While few studies correlate BP parameters and structural damage to the optic nerve head [13], few have studied the same in glaucoma suspects.

This study aimed to compare the 24-hour BP pattern in glaucoma suspects and determine the relationship between Retinal Nerve Fibre Layer (RNFL) thickness and diurnal variation in BP, more importantly, nocturnal dip.

## Materials and methods

### Study setting

After obtaining institutional ethics committee approval, we conducted this cross-sectional descriptive study in a tertiary health center in South India from July 2014 to June 2016.

### Patient selection

Consecutive cases of glaucoma suspects from the glaucoma clinic and cases undergoing cataract surgery from the cataract clinic were selected for the study. The Institute Ethics Committee (IEC) - Human Studies approved the study protocol and followed the guidelines as per the Declaration of Helsinki. Informed consent was taken from the participants in their native language.

The study was comprised of two groups of patients above the age of 40. The study group consisted of glaucoma suspects and was defined as gonioscopically open angles with one among the following in at least one of the eyes: consistently elevated IOP > 21 mm Hg by Goldmann Applanation Tonometry (GAT) or optic nerve head or retinal nerve fiber layer (RNFL) appearance suggestive of glaucomatous damage, diffuse or focal narrowing or sloping of the disc rim, disc hemorrhage, diffuse or localized abnormalities of the RNFL, or visual fields suspicious of early glaucomatous damage [14].

The control group consisted of age and gender-matched patients with IOP < 21 mm Hg, a typical optic disc picture, and no family history of glaucoma. They were recruited from the set of patients admitted for cataract surgery. Those with evidence of intracranial lesions, history of massive hemorrhage or hemodynamic crisis, previous or current use of anti-glaucoma medications, any other ophthalmic and neurologic disease that could result in VF defects, history of diabetes mellitus, and history of hypertension taking medications other than calcium channel blockers (CCB) and beta blockers were excluded from the study.

The primary outcome measures were 24-hour blood pressure readings and retinal nerve fiber thickness assessment. The secondary outcome measure was the correlation between nocturnal dip and average nerve fiber thickness.

### Retinal nerve fiber layer assessment

RNFL assessment was done using optical coherence tomography (OCT) (Stratus OCT, Carl Zeiss, USA) and the FAST RNFL scan protocol. A signal strength of 7 or more was considered a good scan and was taken for analysis. The parameters assessed were average, maximum, and minimum RNFL thickness.

### 24-hour ambulatory blood pressure (ABP) monitoring

**Fig. 1:**
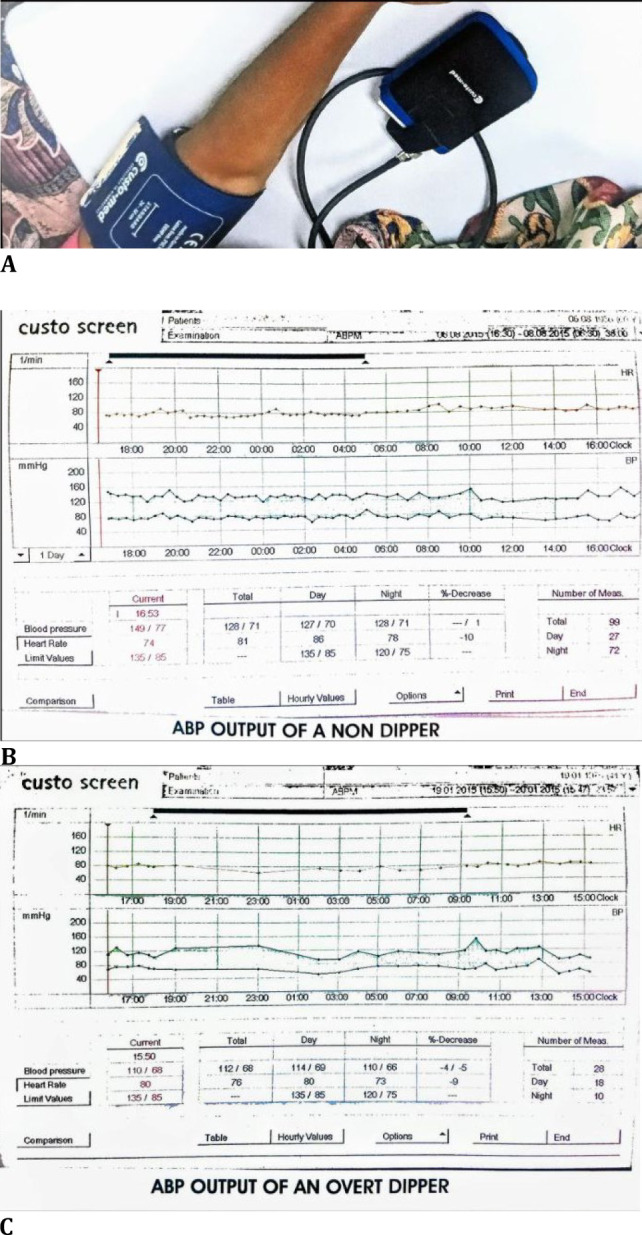
**A**. ABP Monitoring, **B**. ABP Output of a Non-Dipper, **C**. ABP Output of an Overt Dipper

Patients in both groups underwent in-hospital measurement of 24hr ABP every 30 minutes during the day time (5 am-5 pm) and every 20 minutes during the night (5 pm-5 am) using an ambulatory BP monitoring device (CustoMed, GMBH, Germany) (**Fig. 1 A**). This measured the BP recordings under physiological conditions without any inter-observer bias. The various parameters recorded were mean systolic BP (SBP), mean diastolic BP (DBP), mean SBP during the day and that during the night, similarly mean DBP during the day and that during the night, mean arterial pressure (MAP) and the nocturnal dip in SBP and DBP (**Fig. 1 B, C**).

MAP was calculated using the formula:

MAP=DBP+[1/3×(SBP–DBP)]

### Classification of patients: dippers vs. non-dippers

Patients were classified as dippers and non-dippers based on the nocturnal BP reduction values. Patients with < 5% nocturnal BP reduction were classified as non-dippers, and patients with > 5% nocturnal BP reduction were defined as dippers. A dip in nocturnal BP of more than 10% was defined as overt dippers.

### Sample size calculation

The sample size was calculated based on the assumption that approximately 80% of the general population shows a physiologic nocturnal dip in systemic BP. Maintaining a precision of 10% and a possible dropout rate of 10% and assuming a prevalence of 80% dippers among the general population, the sample size was calculated to be 100 [15].

### Statistical analysis

All data were expressed as mean ± standard deviation. An unpaired t-test was used to compare the blood pressure data between the two groups. ANOVA and Post Hoc test (Bonferroni) analysis were performed to analyze RNFL thickness between non-dippers, dippers, and overt dippers among glaucoma suspects. Pearson’s correlation assessed the relationship between the nocturnal dip and average RNFL thickness. All statistical tests were done at a 5% significance level, and a p-value < 0.05 was considered statistically significant. All statistical analyses were performed using IBM PASW Statistics (SPSS) version 19 (IBM Corp., Armonk, NY, USA).

## Results

A total of 200 patients were included in the study, 100 each in glaucoma suspect and control groups. The mean age of the glaucoma suspect group was 59.57 ± 5.70 years (range from 43 to 71 years), and that in the control group was 61.20 ± 4.27 years (range from 52 to 71 years). 105 male (53 controls and 52 cases) and 95 female (47 controls and 48 cases) patients participated in the study.

The mean daytime and night-time SBP, mean SBP, mean daytime and night-time DBP, mean DBP, and MAP in both groups are shown in **Table 1**. Glaucoma suspect group showed statistically significant low SBP(D), SBP(N), and mean SBP compared to the controls. However, the DBP values (DBP(D), DBP(N), and mean DBP) were higher among glaucoma suspects, which was found to be statistically significant. There was no statistically significant difference regarding the MAP between the groups.

The mean nocturnal blood pressure dip was higher in glaucoma suspects (6.53 ± 2.42 mm Hg) than in the control group (6.41 ± 2.12 mm Hg) studied. However, the difference was not statistically significant (**Table 1**).

**Table 1:** Comparison of different BP parameters among cases and controls

BP Parameters	CONTROL GROUP ^a^	CASE GROUP ^a^	p VALUE
SBP(D)	139.10 ± 7.59	136.00 ± 7.34	0.004**
SBP(N)	132.60 ± 6.71	129.29 ± 7.26	0.001***
MEAN SBP	135.80 ± 7.05	132.65 ± 7.14.	0.002**
DBP(D)	79.03 ± 6.03	82.65 ± 5.35	0.001***
DBP(N)	72.73 ± 6.33	76.16 ± 5.63	0.001***
MEAN DBP	75.94 ± 6.02	79.41 ± 5.25	0.001***
MAP	95.87 ± 5.79	97.15 ± 4.53	0.091
MEAN NOCTURNAL DIP	6.41 ± 2.12	6.53 ± 2.42	0.712

(^a^n = 100 in both case and control group, all BP values are expressed in mm Hg. All data is expressed as mean ± standard deviation DBP = Diastolic BP; DBP (D) = Mean Day-time DBP; DBP (N) = Mean Night-time DBP MAP = Mean Arterial Pressure SBP = Systolic BP; SBP (D) = Mean Day-time SBP; SBP (N) = Mean Night-time SBP)

**Fig. 2:**
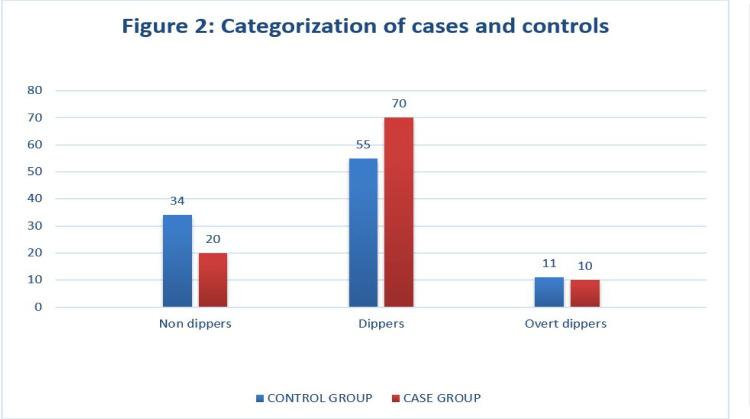
Categorization of cases and controls as Non-Dippers, Dippers, and Overt Dippers

We found that 80 out of the 100 glaucoma suspects showed a nocturnal dip in BP compared to 66 out of the 100 participants in the control group. However, the difference was not statistically significant (**Fig. 2**). The number of non-dippers was higher in controls, and glaucoma suspects included a more substantial number who showed a > 5% dip in nocturnal BP. Both groups showed almost equal overt dippers (10% and 11%, respectively).

The mean average RNFL thickness among glaucoma suspects was 101.15 ± 11.31 µm. The inferior RNFL thickness was more than the superior RNFL (125.15 ± 10.97 µm vs. 123.12 ± 12.46 µm).

ANOVA and Post Hoc test (Bonferroni) analysis of RNFL thickness between non-dippers, dippers, and overt-dippers among glaucoma suspects showed the following results (**Table 2**): overt dippers showed statistically significant thinning of superior, inferior, and average RNFL thickness as compared to non-dippers and dippers; though dippers showed objective thinning of superior, inferior, and average RNFL thickness compared to non-dippers, the difference was not statistically significant.

**Table 2:** RNFL thickness description of non-dippers, dippers, and overt dippers among glaucoma suspects

	NON-DIPPERS	DIPPERS	OVERT DIPPERS
SUPERIOR RNFL (avg.)	128.05 ± 11.293	124.70 ± 9.489	102.20 ± 13.774
INFERIOR RNFL (avg.)	130.10 ± 9.481	126.07 ± 9.341	108.80 ± 10.304
RNFL THICKNESS (avg.)	105 ± 11.183	102.19 ± 9.582	86.20 ± 12.200

(All data is expressed as mean ± standard deviation; RNFL thickness is expressed as µm)

**Fig. 3:**
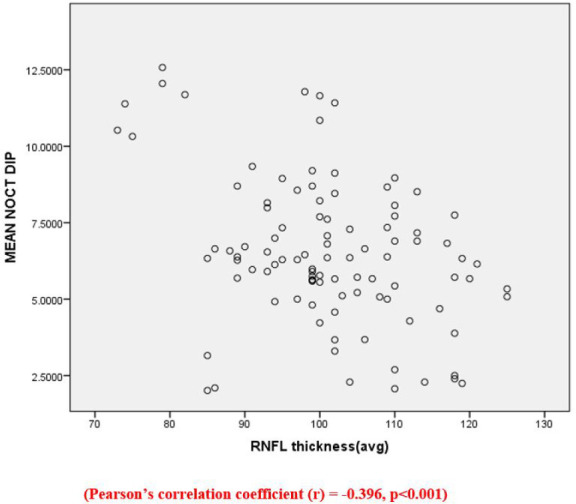
Scatter plot between mean nocturnal dip in blood pressure and average RNFL thickness

The scatter plot (**Fig. 3**) shows a negative correlation between nocturnal dip in BP and RNFL thickness, i.e., a decrease in RNFL thickness with an increase in mean nocturnal dip in BP (Pearson’s correlation coefficient r = -0.396, p < 0.001).

## Discussion

Studies have yet to be done among patients with POAG and NTG to establish vascular etiology in glaucomatous optic nerve damage pathogenesis [11,12,16,17]. In this study, the blood pressure parameters over 24 hours were recorded using the ambulatory monitoring method. We assessed the daytime and night systolic and diastolic pressures, the nocturnal dip, and the correlation with RNFL thickness.

The key findings of this paper were the pronounced lower systolic BP and high diastolic BP at both daytime and nighttime in glaucoma suspects compared to the healthy population, which was statistically significant. Glaucoma suspects had a much higher proportion of subjects with nocturnal BP dipping compared to healthy controls, which was, however, not important. The overt dippers significantly reduced the RNFL thickness compared to non-dippers and dippers. Furthermore, a weak negative correlation existed between nocturnal dip and RNFL thickness.

In the general population, about 2/3rd of the people show a physiological dip at night in systemic blood pressure of about 5-10%, while the remaining are either non-dippers or overt dippers [16]. As ocular perfusion pressure (OPP) depends on blood pressure and intraocular pressure, changes in blood pressure are indicated by altering the perfusion to ONH. A deranged auto-regulation mechanism further alters the ocular perfusion. This variation in BP, especially the physiological nocturnal dip because of the prone position, is postulated in the pathogenesis of ischemic optic nerve damage.

Glaucoma suspect group showed statistically significant low SBP(D), SBP(N), and mean SBP compared to the controls. In their study, Kaiser et al. showed a similar pattern of lower day and night SBP in subjects with POAG with worsening glaucomatous damage despite a good IOP control profile and in subjects with NTG [11].

However, the DBP values (DBP(D), DBP(N), and mean DBP) were significantly higher among glaucoma suspects. The Beaver Dam Eye Study showed that a 10 mm Hg increment of DBP can elevate the IOP by 0.43 mm Hg, while the Rotterdam Study showed this increment to be 0.23 mm Hg [3,6]. Kaiser et al. found no significant difference in the DBP values when comparing their subjects with controls [11].

There was no statistically significant difference regarding the MAP between the groups. In their study, Graham et al. also showed that the mean blood pressure values did not differ significantly; the fluctuation and nocturnal BP dips were associated with glaucoma progression [12].

Nocturnal dip in BP is implicated in the pathogenesis of open-angle glaucoma and NTG. Previous studies have been conducted primarily on normal tension glaucoma to study the 24-hour blood pressure pattern. To mention a few, Meyer et al. showed that subjects with NTG have significant dips in their BP at night compared to controls [9]. In their study done in untreated NTG subjects, Choi et al. showed that night-time dips in BP significantly affected the ocular perfusion pressure, which may impact their visual fields [16]. Moreover, Joe et al. established that nocturnal dips in BP correlated with structural RNFL damage in subjects with untreated NTG [17]. Graham et al. investigated the 24-hour blood pressure pattern in POAG and NTG patients. They showed that subjects with more significant reductions in their nocturnal BP were more prone to have worsening visual fields, even with well-controlled IOP [12]. Kaiser et al. showed that patients with POAG had clinical worsening despite reasonable IOP control, and NTG patients showed significantly lesser day and night SBP [11]. In our study, 80% of glaucoma suspects showed a nocturnal dip in BP compared to 66% in the control group—these observations aligned with the previous studies mentioned in the literature [16,17].

The proportion of patients showing overt dipping patterns was similar in glaucoma suspects (10%) and controls (11%). The prevalence of dippers and overt dippers among glaucoma patients needs to be better established, as no clear definition is stated in the literature. Moreover, various studies on glaucoma patients have also used multiple definitions to categorize the patients as dippers and overt dippers. Hence, comparing our study results with previously available literature is difficult.

The various RNFL parameters assessed were the superior, inferior, and average RNFL thickness, and the values were approximately the same as described by Ramakrishnan et al. for Indian people’s eyes. Although the study mentioned above showed that the RNFL thickness in their study population (Southern India) did not follow the ISNT rule, our study values in a similar population followed the ISNT rule values according to which inferior RNFL is generally considered to be thicker than superior RNFL [18]. We studied the implication of a nocturnal dip in blood pressure with structural optic nerve damage by studying the RNFL thickness. We found a weak negative association between the parameters using correlation studies. This states that a dip in nocturnal blood pressure is associated with thinning of RNFL. This is the most crucial finding of our research, and it points towards vascular etiology in the progression of glaucomatous optic neuropathy.

The strength of our study is that it is one of the few studies to correlate BP parameters and structural damage to the optic nerve head using RNFL thickness in glaucoma suspects. Moreover, the study was adequately powered to generalize the results. Our study, though cross-sectional, had an age and gender-matched control group as a comparator, which also belonged to the same geographical distribution. Hence, the study results can be extrapolated to the target population.

The study’s shortcoming is that we did not consider IOP factors influencing the Ocular perfusion pressure (OPP). As systemic BP is not the only factor associated with OPP, we cannot strongly point out that a dip in BP is the cause of the progression of ONH damage. Hence, there may be other factors too that may contribute to the progression of glaucomatous optic neuropathy. The study has a cross-sectional design, which limits its ability to establish causality. Long-term prospective studies would be needed to determine the causal relationship between nocturnal blood pressure dip and glaucoma progression.

## Conclusion

Our study has strengthened the previously available knowledge that autonomic and vascular dysregulation has a role in the pathogenesis and progression of optic neuropathies. Including BP monitoring in the workup of a glaucoma suspect will help in the early detection of patients who are likely to progress to primary open-angle glaucoma and earlier treatment may be provided. However, further long-term prospective studies are needed to assess the possibilities of incorporating systemic vascular evaluation in the workup of a glaucoma suspect.
